# Highly temporal stable, wavelength-independent, and scalable field-of-view common-path quantitative phase microscope

**DOI:** 10.1117/1.JBO.25.11.116501

**Published:** 2020-11-11

**Authors:** Azeem Ahmad, Vishesh Dubey, Ankit Butola, Balpreet Singh Ahluwalia, Dalip Singh Mehta

**Affiliations:** a UiT The Arctic University of Norway, Department of Physics and Technology, Tromsø, Norway; b Indian Institute of Technology Delhi, Department of Physics, Hauz Khas, New Delhi, India

**Keywords:** quantitative phase imaging, single-shot multi-wavelength imaging, scalable FOV and common path interferometry

## Abstract

**Significance:** High temporal stability, wavelength independency, and scalable field of view (FOV) are the primary requirements of a quantitative phase microscopy (QPM) system. The high temporal stability of the system provides accurate measurement of minute membrane fluctuations of the biological cells that can be an indicator of disease diagnosis.

**Aim:** The main aim of this work is to develop a high temporal stable technique that can accurately quantify the cell’s dynamics such as membrane fluctuations of human erythrocytes. Further, the technique should be capable of acquiring scalable FOV and resolution at multiple wavelengths to make it viable for various biological applications.

**Approach:** We developed a single-element nearly common path, wavelength-independent, and scalable resolution/FOV QPM system to obtain temporally stable holograms/interferograms of the biological specimens.

**Results:** With the proposed system, the temporal stability is obtained ∼15  mrad without using any vibration isolation table. The capability of the proposed system is first demonstrated on USAF resolution chart and polystyrene spheres (4.5-μm diameter). Further, the system is implemented for single shot, wavelength-independent quantitative phase imaging of human red blood cells (RBCs) with scalable resolution using color CCD camera. The membrane fluctuation of healthy human RBCs is also measured and was found to be around 47 nm.

**Conclusions:** Contrary to its optical counterparts, the present system offers an energy efficient, cost effective, and simple way of generating object and reference beam for the development of common-path QPM. The present system provides the flexibility to the user to acquire multi-wavelength quantitative phase images at scalable FOV and resolution.

## 1 Introduction

Quantitative phase microscopy (QPM) is a powerful technique, which works on the principle of interferometry.^1^ QPM has the capability to measure the phase maps and subsequently the refractive index and dry mass density of the biological cells.^1^^–^^3^ To produce interference, there must be at least two coherent beams, one reference and one object beam. In the past, several attempts have been made by many researchers to produce two coherent beams. In most of the QPM systems, i.e., Michelson,^4^ Linnik,^5^^–^^9^ Mach–Zehnder,^10^ and Mirau, interferometer geometries^2^^,^^11^^–^^13^ are generally employed to produce two coherent beams, one from the reference arm and other from the object arm. These optical configurations for QPM, being non-common path in nature, suffer from several serious problems: time-varying phase noise due to vibration, temperature gradient, and air flow, which deteriorate the stability of QPM.^3^^,^^14^ These issues limit the application of QPM systems for the study of live cells dynamics, i.e., measurement of membrane fluctuations, which is a good indicator for the detection of several diseases.^14^

During the last decade, various common-path QPM techniques have been developed to minimize the temporal phase noise.^1^^,^^3^^,^^14^^–^^18^ Diffraction phase microscopy^3^^,^^14^^,^^19^ or spatial light interference microscopy^15^ employs diffraction grating and SLM, which makes the system costly. The diffraction grating diffracts the input beam into several orders, which reduces the diffracted beam intensity into zero and +1 order by several orders of magnitude. The diffracted intensity into these two orders can be enhanced by employing blaze grating, which diffracts most of the incident light into either the zero or positive first order at the cost of 4% reflection from the grating surface and very little diffracted light into the negative first and higher orders.^3^ However, diffraction grating limits the use of the incident light wavelength. It is optimized only for a particular wavelength for which it diffracts the maximum optical power into a specified diffraction order. Moreover, the grating pitch decides the parameters [magnification and numerical aperture (NA)] of the objective lens to be used. To make the technique fully functional, ∼3 to 4 grating lines should pass through the airy disk produced by the objective lens.^3^ The diffraction grating is thus in-directly coupled to the chosen objective lens of given magnification and resolution. Therefore, if the objective lens is changed with different magnifications or NA, the diffraction grating must be changed accordingly to achieve optimal performance of the system.^14^ This increases the complexity, i.e., the need of realignment, and the practicality of the system.

In 2012, lateral shearing digital holographic microscopy (DHM) technique is also developed to achieve high temporal stability.^16^ However, this technique also has its own limitation, e.g., amount of shear between the reflected wave fronts generated due to the front and back surface of the shear plate has to be greater than the size of the object under study. Therefore, it cannot be implemented for closely spaced biological or industrial objects, i.e., sample has to be spatially dispersed. Further, the technique presented in Ref. 16 utilizes only 4% to 8% intensity of the input beam. To overcome these challenges a simple, comparatively energy efficient and cost-effective method is required.

In this work, a highly stable QPM system is developed to overcome aforementioned issues. Two nearly common-path coherent beams are generated with the combination of 6-mm-thick glass plate and pinhole assembly, thus provides high temporal stability to the QPM system. The glass plate has ∼20% front and ∼100% back reflection surfaces coating. The back surface reflected beam, which carries high intensity, is passed through 30  μm pinhole to generate the reference beam from the object beam. The temporal stability of the proposed system is measured to be equal to ∼15  mrad without using vibration isolation table. The experiments are conducted on USAF resolution target, polystyrene sphere (4.5-μm diameter), and human red blood cells (RBCs). Further, wavelength-independent and scalable field of view (FOV) QPI is performed with the proposed system. The membrane fluctuation of healthy RBCs is then quantified and found to be greater than the system’s temporal stability (∼15  mrad). The standard deviation (SD) of human RBC’s membrane fluctuation is measured to be equal to 47 nm. The newly developed phase microscope can be easily integrated with other dynamical behavior studying techniques.^5^^,^^20^^,^^21^

## 2 Highly Stable Common-Path Quantitative Phase Microscope

**Fig. 1:**
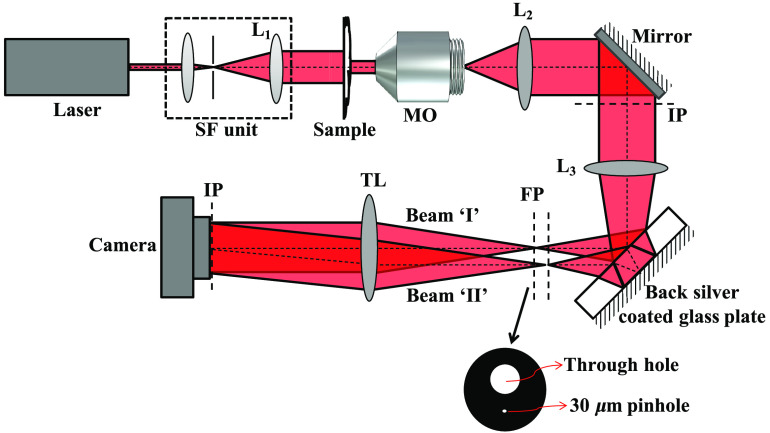
The schematic depiction of the transmission-mode common-path digital holographic quantitative phase microscope for biomedical imaging. SF unit, spatial filtering unit; MO, microscope objective; and IP, image plane. The amplitude mask having through-hole (dia. 6 mm) and pinhole (dia. 30  μm) situated at Fourier transform plane.

A highly stable, comparatively energy efficient and cost effective common-path quantitative phase microscope is illustrated in Fig. 1. In the present technique, a way for generating the reference beam and the object beam of approximately equal intensity is presented. This is achieved using a combination of back surface silver-coated optical glass plate and a pinhole assembly. The highly coherent light beams generated by Cobolt Flamenco™ (at 660 nm) and diode pumped solid state (DPSS) (at 532 nm) laser sources are passed through a spatial filtering unit to generate a clean beam. Two different lasers are not shown in the schematic of the experimental setup. The spatially filtered diverging beam is further made collimated by employing a collimating lens $L_{1}$ into the beam path. Thus the sample is illuminated from a collimated light beam, which avoids inaccuracy in phase measurement of the specimen. The transmitted light through the specimen is finally collected by microscope objective (MO) lens, which is further imaged at the image plane (IP) using a lens $L_{2}$.

The object beam coming from the microscopic imaging system is then converged at Fourier plane (FP) by inserting a condenser lens $L_{3}$. The coated glass plate (20% front and 100% back surface) is placed into the beam path that generates multiple beams due to multiple reflections between the front and the back surfaces of the glass plate. Each beam has the intensity of 20% of the previous beam. The first reflection coming from the back surface of the glass plate has high intensity compared to the other beams. The high-intensity beam and the front reflection from the glass plate are further passed through a custom designed metal disk having two through holes.

In one of the hole, a pinhole of 30-μm diameter is inserted. This pinhole containing disk is placed into the beam path in a way such that high-energy beam should pass through the pinhole, which blocks most of the intensity (∼70%) of the beam, thus generating a reference beam of approximately the same intensity as that of object beam (20% reflection from the front surface). After the pinhole plane, there are only two beams (object and reference) reaching at the camera. Other beams generated due to the multiple reflections in the glass plates have very less intensity and are blocked by the pinhole holder. The cost to fabricate the back surface-coated glass plate is around 1 $ or less. The cost of the pinhole plate is also around 1 $. In total, the cost of both the components is around 2 $ or less. These two beams having almost equal intensity recombine at the detector plane using tube lens (TL) to generate high-contrast spatially modulated interferograms of the specimen under study.

The spatially modulated interferograms, generated due to the superposition of spatially filtered beam “II” (after passing through pinhole) and unfiltered beam “I,” are further processed through the Fourier transform-based phase retrieval algorithm^22^ to measure the phase shift introduced by the specimen. The measured phase shift is related to the refractive index and height of the sample as

$$\Delta \varphi (x,y)=\frac{2\pi }{\lambda }\Delta nh(x,y),$$ (1)

where λ is the illumination wavelength, Δn is the refractive index difference between the sample and surrounding medium, and h(x,y) is the sample thickness. The recovered phase map contains the coupled information of the refractive index and the thickness of the specimens. The thickness of the specimens can be measured if the refractive index is known or vice versa.

The coated glass plate, which is used to generate two coherent beams, introduces an axial focal shift at the pinhole plane as a function of wavelength due to the dispersion effect of the glass plate. However, the amount of the axial shift will not be very large (not greater than the depth of field of the lens $L_{3}$), when the light beam is switched from 532 to 660 nm wavelength. There would be a small axial shift, which does not influence the spatial filtering for the reference arm adversely. In order to understand this, a systematic calculation is done. First, we calculated the depth of field of the 75-mm focal length lens. The depth of field of the lens is calculated to be around 60  μm. The axial focal shift due to glass plate for 532 and 660 nm is calculated to be around 30  μm when a glass plate of 6-mm thickness at 45 deg is inserted in the light beam path (after the lens) as shown in Fig. 1. Thus the axial focal shift is within the depth of field of lens L3. Therefore, there would not be a significant effect of the dispersion due to the glass plate on the spatial filtering for the reference beam. Thus the proposed set-up enables simultaneous multi-wavelength phase imaging with scalable resolution as later demonstrated. The scalable resolution comes from easy of changing the objective lens without needing to realign the optical set-up.

## 3 Results and Discussion

### 3.1 Temporal Phase Stability

In order to illustrate the temporal stability of the present system, a series of time lapsed interferograms having size 512×512  pixels without any sample were recorded (Video S1). Video S1 exhibits the comparison of a temporally unstable (based on Linnik interferometer)^8^ and stable (present approach) QPM system. The time lapsed interferograms recorded with the present design were further processed to measure the temporal fluctuation of phase over the whole FOV. The temporal phase fluctuation of the system is calculated as

Δϕ(x,y,t)=ϕ(x,y,t)−ϕ(x,y,t=0). (2)

**Fig. 2:**
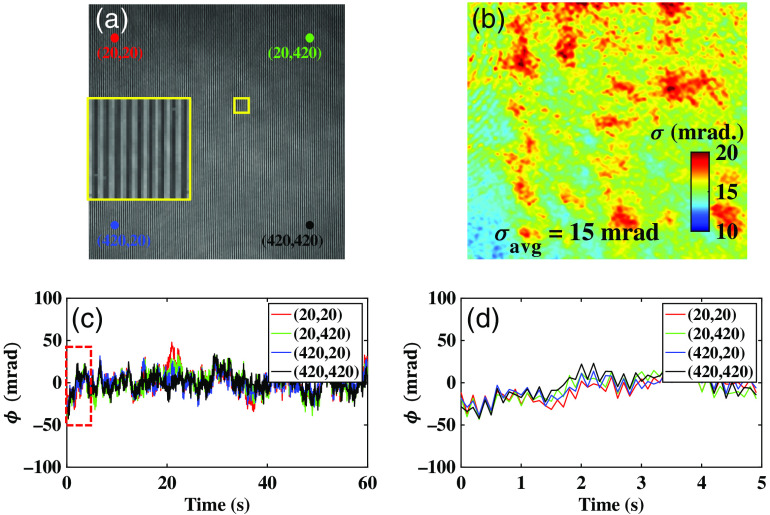
Temporal phase stability measurement of developed phase microscope under ambient environmental fluctuation. (a) Sample free interferogram generated due to the superposition of object and reference beam. (b) Pixel by pixel variation of SD of the temporal phase fluctuation over the whole FOV. (c) Temporal variation of phase as a function of time at four different locations (20, 20), (20, 420), (420, 20), and (420, 420) of the interferogram. (d) Zoomed view of the region marked with red dotted box in Fig. 2(c) to clearly illustrate the variation of phase as a function of time. Comparison of a non-common path (temporally unstable) and common path (temporally stable) interferometer. The non-common path interferometer works on the principle of Linnik interferometer and common path interferometer is the proposed optical scheme (Video S1, mov, 1 MB [URL: https://doi.org/10.1117/1.JBO.25.11.116501.1]).

In order to check the temporal sensitivity behavior across the full FOV, the temporal phase fluctuation is measured at four different locations (20, 20), (20, 420), (420, 20), and (420, 420) of the interferogram as shown in Fig. 2(a). Figure 2(b) depicts the pixel by pixel variation of SD of the temporal fluctuation over the whole FOV under the ambient environmental fluctuations.^23^ The average temporal fluctuation is measured to be approximately equal to 15 mrad. The slight variation in the behavior of the temporal noise is observed across the whole FOV as illustrated in Fig. 2(b). This could be due to the detector’s noise, light source power instability, and coherent noise generated due to the laser light source. Figure 2(c) illustrates the fluctuation of the recovered phase at four different locations (20, 20), (20, 420), (420, 20), and (420, 420) as a function of time. The zoomed view of region marked with red dotted box of the phase fluctuation is depicted in Fig. 2(d).

### 3.2 USAF Resolution Test Target

**Fig. 3:**
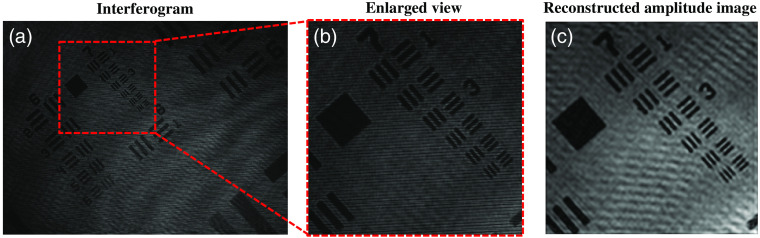
(a) Fourier hologram of USAF resolution chart captured using the present common-path QPM. (b) Enlarged view of the small portion of the interferogram of Fig. 3(a) enclosed by red dotted box. (c) Reconstructed amplitude image of resolution target obtained using Fourier transform method.

The developed DHM system is, first, tested by performing experiments on USAF resolution test target. A monochrome camera (Lumenera—Infinity2-1R) and MO (60×/0.7  NA) is used to image the test target. The USAF chart is placed into the beam path to record the corresponding high fringe density interferogram as illustrated in Fig. 3(a). Figure 3(b) depicts the enlarged view of the region marked with red dotted box shown in Fig. 3(a). The recover amplitude information of the resolution chart is presented in Fig. 3(c). In order to measure the transverse resolution of the system, we implemented slant edge method.^24^ The spatial resolution of the system is measured to be equal to 765 nm.

### 3.3 Quantitative Phase Imaging

#### 3.3.1 Polystyrene spheres

To test the phase measurement accuracy of the present system, an experiment is done on the polystyrene spheres (Polysciences, Inc. Catalog # 17135) with diameter of 4.5±0.152  μm and refractive index of 1.59 at 632 nm wavelength. The polystyrene spheres are first washed 2× with distilled water and then immersed in ethanol. A small volume of polystyrene solution pipetted into a $3\times 3\;{\mathrm{mm}}^{2}$ polydimethylsiloxane chamber attached with microscopic glass slide and left for 5 to 10 min for the evaporation of ethanol. Then a drop of immersion oil (RI∼1.51) is put onto the dried polystyrene sphere’s monolayer to avoid the lensing effect caused by its spherical nature. The sample is sealed from the top with a 170-μm thick cover glass.

**Fig. 4:**
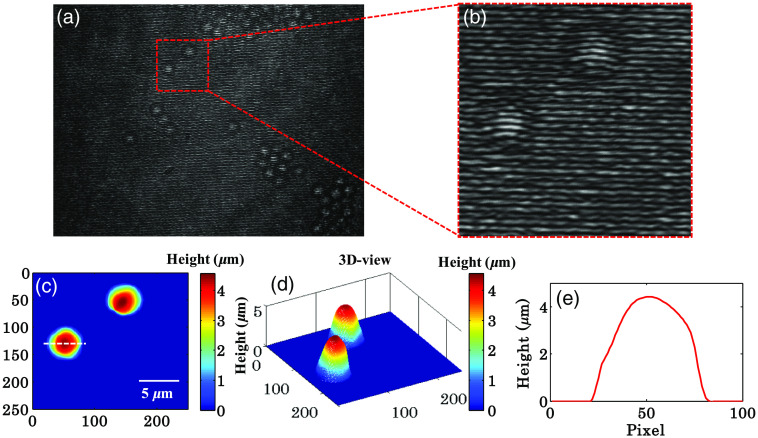
(a) Full-frame interferometric image of polystyrene spheres (dia. ∼4.5±0.152  μm) captured using the present common-path QPM. (b) Enlarged view of the small portion of polystyrene spheres interferogram of Fig. 4(a) enclosed by red dotted square. (c)–(e) 2D-view, 3D-view, and corresponding line profile (along white dotted line) of the height map of polystyrene spheres obtained from phase map using Fourier transform method. Color bar shows the height in μm.

The sample is placed under the developed QPM for phase recovery and subsequently height measurement of polystyrene spheres. For poly bead imaging, again monochrome camera (Lumenera—Infinity2-1R) and MO (60×/0.7  NA) is used. Figure 4(a) illustrates full-frame interferogram of polystyrene spheres generated by the present setup. The magnified view of the region marked with red dotted box is depicted in Fig. 4(b) and subsequently utilized to measure phase/height map of polystyrene spheres. Figures 4(c) and 4(d) exhibit the 2D and 3D views of the measured height map of the polystyrene spheres. The refractive indices of polystyrene spheres and outside medium (immersion oil) are considered to be equal to 1.59 and 1.51 at 632 nm wavelength for the calculation. The height profile of the polystyrene sphere along the white dotted line shown in Fig. 4(c) is depicted in Fig. 4(e). The height of the polystyrene spheres is measured to be equal to 4.42  μm, which is found to be in a good agreement with the values (∼4.5  μm) provided by the manufacturer.

#### 3.3.2 Membrane fluctuation of human RBCs

To exhibit the potential application of high temporal stability of the proposed QPM system, membrane fluctuation of the healthy RBC is measured. Fresh blood sample is collected from the hospital in the ethylenediaminetetraacetic acid containing tubes, which avoids the coagulation of RBCs. The blood sample was then diluted using phosphate buffer saline (PBS) and washed 2× to isolate the human RBCs from the rest of the blood components such as white blood cells, platelets, and plasma. Further, sample was prepared on a microscopic glass slide and placed under the microscope for the measurement of RBC’s membrane fluctuation.

**Fig. 5:**
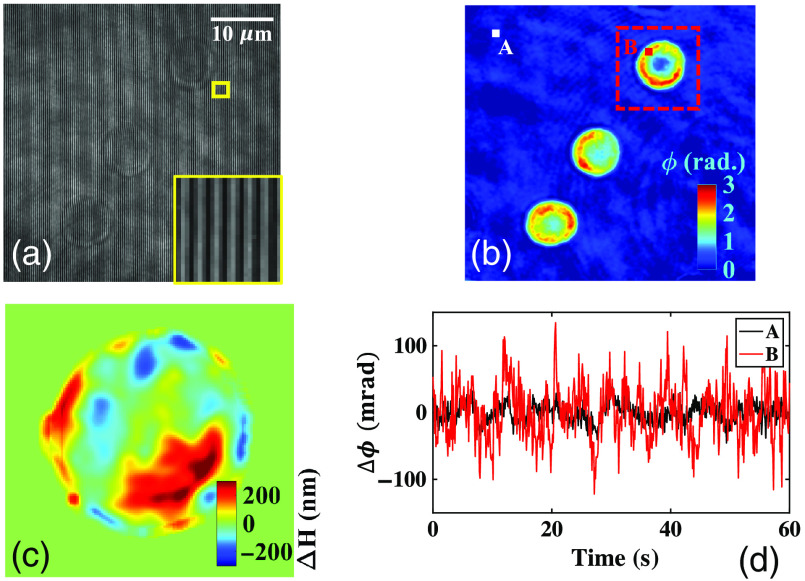
Membrane fluctuation measurement of healthy RBC. (a) One of the interferogram of human RBCs extracted from time lapsed interferometric movie. (b) Corresponding recovered phase map. (c) Respective instantaneous displacement map (color bar in nm) (see Video S2). (d) Temporal fluctuation of the recovered phase at point A (background) and point B (RBC) as a function of time [Fig. 5(b)]. Time-lapsed interferometric movie of human RBCs at the frame rate of 100 fps. Corresponding temporal variation of the phase map and displacement map of the human RBCs (Video S2, mov, 2.4 MB [URL: https://doi.org/10.1117/1.JBO.25.11.116501.2]).

To assess the RBC’s membrane fluctuation measurement capability of the proposed system, we acquired a 1-min time-lapsed interferometric movie at 100 fps of human RBCs under ambient environmental fluctuations and without vibration isolation table (see Video S2). Each interferometric frame of the movie is processed by employing Fourier transform-based phase recovery algorithm for the measurement of phase/height fluctuations of RBCs. The refractive index of human RBC and outside medium (PBS) are considered to be equal to 1.40 and 1.33, respectively, during the calculation.^25^ Figure 5(a) depicts one of the interferogram of human RBCs extracted from the recorded movie. The corresponding recovered phase map is illustrated in Fig. 5(b). The temporal fluctuation of the RBC’s phase map can be seen in Video S2. For the measurement of membrane fluctuation, first, average height map of RBCs is calculated from the recovered phase images. Then the average height map is subtracted from each instantaneous height map corresponding to each frame of the interferometric movie.

The instantaneous displacement map of the RBC is depicted in Fig. 5(c), which is found to be in a close agreement with the previous results.^25^ The phase changes at two different positions are monitored over 60 s [Fig. 5(d)]. The point on the glass slide (A) exhibits the temporal stability of the QPM system. The SD of height fluctuation at point A is measured to be equal to 15.2 nm. The point chosen on the RBC membrane (B) presents its dynamic fluctuation and the SD is measured to be equal to 47 nm. It is worth highlighting that the present system has the capability to quantify RBC’s dynamic fluctuations under environmental fluctuations, which can be further employed for various disease diagnosis.

#### 3.3.3 Simultaneous wavelength-independent QPM

To show the feasibility of the present system for performing simultaneous wavelength-independent QPM, the monochrome camera is being replaced with the single-chip color CCD camera (Lumenera—Infiniy2-3). Two different lasers (peak wavelengths 532 and 660 nm) and imaging objective lens 60×/0.7  NA are used in the developed system (Fig. 1) to perform dual-wavelength QPI of human RBCs. The single-chip color CCD camera is used to perform this study which suffers from the problem of color cross talk between the RGB color channels of the camera. This is due to the use of soft polymer dyes, which are used to make Bayer color filters of the camera.^26^ The soft polymer dye used to make red color filter does not completely block the green wavelength or vice versa. Thus the red filter always transmits some of the photon corresponding to green wavelength or vice versa and leads to the generation of color cross talk between the color channels of the camera.

**Fig. 6:**
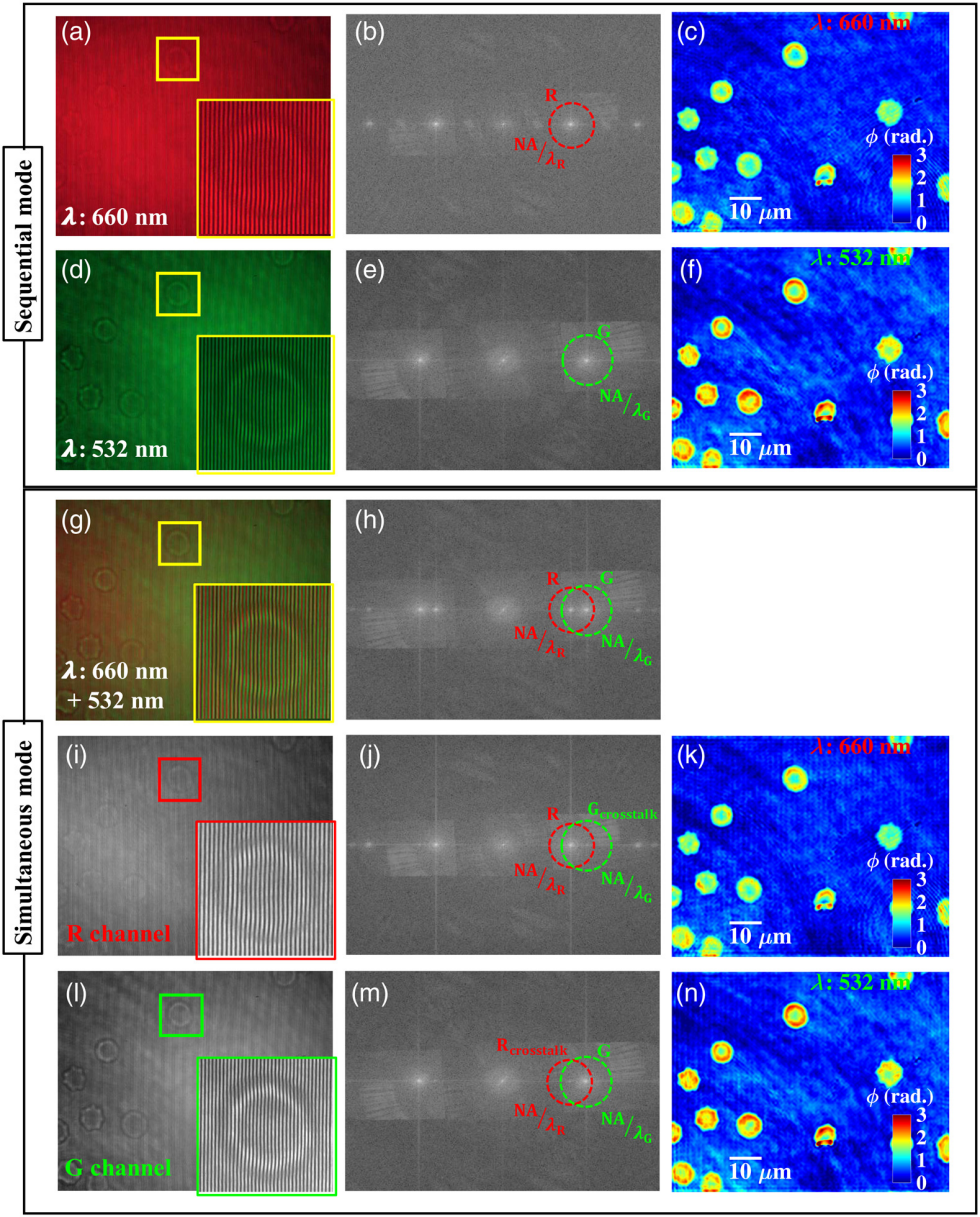
Sequential and simultaneous recording of dual wavelength interferogram and their comparison. (a), (d) Sequentially recorded full-frame interferometric image of human RBCs using color CCD camera at 660 and 532 nm wavelengths, respectively. (b), (e) Corresponding Fourier spectrums and (c), (f) recovered phase maps. (g), (h) Simultaneously recorded full-frame interferogram of human RBCs using color CCD camera at 660 and 532 nm wavelengths and corresponding Fourier spectrum. (i), (l) Numerically decomposed red and green color channel of the dual-color interferogram. (j), (m) Corresponding Fourier spectrums and (k), (n) recovered phase maps. The insets depict the zoomed views of the regions marked with yellow, red, and green color boxes. Color bars show the phase in rad. (Scale bars: 10  μm).

A systematic study is done to understand the effect of color cross talk on the recovered phase maps of the specimens. First, 660 and 532 nm lasers are sequentially switched on and corresponding interferograms of RBCs are recorded by the color camera as illustrated in Figs. 6(a) and 6(d). Figures 6(b) and 6(e) represent the Fourier spectrums of the recorded interferograms. The green and red dotted circles with radius $\mathrm{NA}/\lambda_{R}$ and $\mathrm{NA}/\lambda_{G}$ illustrate the observable regions corresponding to the 660- and 532-nm wavelengths interferograms, which encompasses the range of spatial frequencies of the specimens collected by the imaging objective lens. The recovered phase maps of the human RBCs corresponding to 660 and 532 nm wavelengths are depicted in Figs. 6(c) and 6(f), respectively.

Next, both lasers are switched on simultaneously to acquire the single-shot color multiplexed interferogram for both wavelengths as shown in Fig. 6(g). The color multiplexed interferogram is decomposed into the red and green color channel using MATLAB as shown in Figs. 6(i) and 6(l). These color decomposed interferograms are further processed to recover the simultaneous dual-wavelength phase maps of human RBCs as shown in Figs. 6(k) and 6(n).

It can be clearly visualized that the recovered phase maps of RBCs suffer from the unwanted background modulation in case of simultaneous recording of the interferograms at 660 and 532 nm wavelengths. This is due the presence of color cross talk between the color channels of the camera. The presence of color cross talk can be clearly visualized in the Fourier spectrum of color decomposed interferograms as illustrated in Figs. 6(j) and 6(m). There could be two possible ways to remove the unwanted background modulation generated due to the color cross talk from the recovered phase images. First, the background modulation can be removed by blocking the dominant spatial frequency components corresponding to the color cross talk ($G_{\text{crosstalk}}$ and $R_{\text{crosstalk}}$) in the frequency domain at the cost of slight loss of the object information. Second, the problem of background modulation can also be removed using three-chip CCD camera instead of one-chip color CCD camera.^26^ Thus the method can be implemented for highly stable, multi-wavelength QPM of biological specimens.

#### 3.3.4 Scalable field of view and resolution

**Fig. 7:**
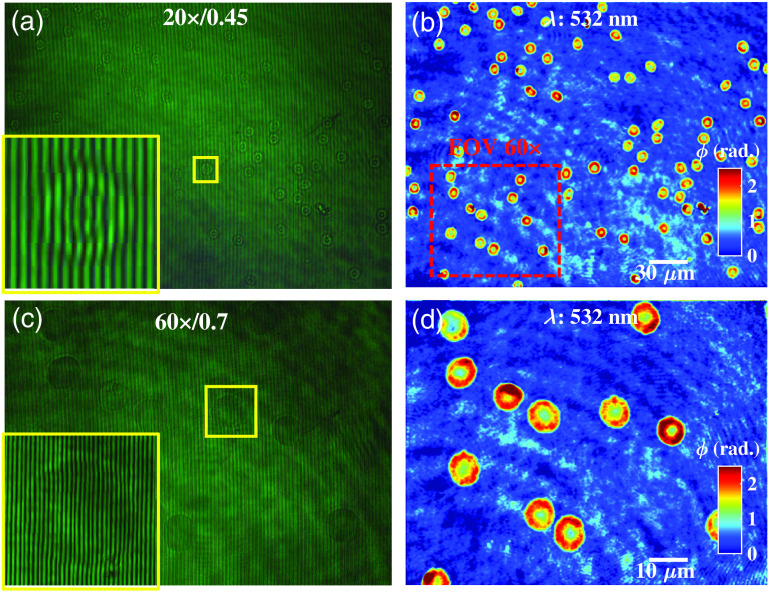
(a), (c) Full-frame interferometric images of human RBCs corresponding to two different MOs 20×/0.45  NA and 60×/0.7  NA, respectively. The insets depict the zoomed views of the regions marked with yellow color boxes. (b), (d) Corresponding full FOV phase reconstruction of human RBCs. The region enclosed by red dotted box in Fig. 7(b) represents the FOV of 60×/0.7  NA objective lens. Color bar shows the phase in rad. The scale bars are in μm.

Next, we demonstrated that the proposed QPM geometry is independent to the choice of imaging objective lens, opening avenues to acquire images with scalable FOV and resolution. Scalable FOV makes the technique viable for various biological applications, where large areas have to be scanned to find the region of interest, followed by imaging at higher resolution for events of interest. To demonstrate the capability of the proposed system, two different MOs 20×/0.45  NA and 60×/0.7  NA were sequentially used for QPM of RBCs. Figures 7(a) and 7(c) illustrate the recorded interferograms of human RBCs corresponding to 20× and 60× objective lenses. The inset in yellow box depicts the zoomed view of region marked with yellow color box in the interferogram. The FOVs of the developed phase microscope are $280\times 210\;{\mu m}^{2}$ and $94\times 70\;{\mu m}^{2}$ for 20× and 60× MOs, respectively. The interferograms are further processed for the phase recovery of human RBCs as illustrated in Figs. 7(b) and 7(d). The region enclosed by red dotted box in Fig. 7(b) represents the FOV of 60×/0.7  NA objective lens. The recovered phase images suffer from a background noise, which is due to the coherent noise of the highly coherent laser light source.

## 4 Conclusion and Discussion

The present method exhibits a novel way of generating the reference beam and the object beam of approximately equal intensity from the sample beam after passing through the MO lens. This is achieved using a combination of silver-coated optical glass plate and a pinhole assembly. The surface flatness of λ/2 or λ is sufficient for the coated glass plate. However, it is not necessary to be equal to λ/2 or λ. Even a normal window glass or normal mirror can also do the job at the cost of aberration in the recovered phase images. The aberration due to optical components (glass plate/mirror) can be removed from the recovered phase images simply by subtracting the phase corresponding to the sample free interferogram. This method of postsubtraction of aberration can effectively work due to the high temporal stability of the system. In addition, if one is interested to produce aberration free phase images then the surface flatness of only the front surface of the glass plate could be critical as it produces the object beam. Surface flatness of the back surface of the glass plate is not critical as the second beam coming from the back surface further passes through a pinhole, which cleans the beam to generate the reference beam.

As shown in Fig. 1, two light beams generated by the glass plate focus at two different planes. The two different focuses of the light beams being used to generate interference pattern will only change the shape of the fringes from straight to circular shape. This is due to the superposition of a plane wave (object arm) and spherical wave (reference arm). Since, the back focal plane of the TL coincides with the focus of the light beam reflected from the front surface of the glass, i.e., the object wave. Therefore, the TL generates a collimated object wave, whereas the reference wave has slight curved wavefront. This leads to the generation of quadratic phase aberration in the recovered phase of the specimen. The quadratic phase aberration is removed from the recovered phase maps of the specimens by simply subtracting the phase corresponding to sample free interferogram. The quadratic phase aberration can also be compensated numerically by employing principle component analysis method.^27^ Thus different FPs of the two beams do not introduce any error in the phase measurement accuracy of the system.

The proposed optical configuration is unique and does not require neutral density filter to equalize the intensity of two beams, which makes it energy efficient. The phase noise of system under ambient environmental fluctuations found to be quite low (around 15 mrad) due to its common path nature. This can be further improved by utilizing vibration isolation table, low-noise camera, and power stable light sources. In addition, there is no need of sparse, i.e., spatially dispersed sample due to the use of pinhole at the FP for the generation of reference beam.

The capability of the present system is exhibited by performing experiments on USAF resolution chart; polystyrene spheres and human RBCs. Replying on high temporal stability of the proposed system, the membrane fluctuations of the healthy RBCs are successfully quantified. Due to its common-path nature, the system can also be implemented with other optical functions such as optical tweezers, waveguide trapping, and microfluidics.

The proposed method enables simultaneous multi-wavelength study of the biological specimens. Also the set-up is not fixed to a given system magnification (fixed objective lens). This makes the set-up independent on the choice of objective lens, enabling imaging with scalable FOV and resolution. This will find applications in acquiring phase images over large FOV and also open avenues for high-throughput QPM.

## Code, Data, and Materials Availability

The authors declare the availability of the data used in the research to obtain the results reported in the manuscript upon reasonable request.
